# Plasticity of Mesenchymal Stem Cells from Mouse Bone Marrow in the Presence of Conditioned Medium of the Facial Nerve and Fibroblast Growth Factor-2

**DOI:** 10.1155/2014/457380

**Published:** 2014-12-29

**Authors:** Eudes Euler de Souza Lucena, Fausto Pierdoná Guzen, José Rodolfo Lopes de Paiva Cavalcanti, Maria Jocileide de Medeiros Marinho, Wogelsanger Oliveira Pereira, Carlos Augusto Galvão Barboza, Miriam Stela Mariz de Oliveira Costa, Expedito Silva do Nascimento Júnior, Jeferson Sousa Cavalcante

**Affiliations:** ^1^Laboratory of Experimental Neurology, Health Science Center, State University of Rio Grande do Norte, Avenida Atirador Miguel da Silva Neto, Airport, 59607-360 Mossoró-RN, Brazil; ^2^Laboratory of Molecular Biology, Health Science Center, State University of Rio Grande do Norte, Avenida Atirador Miguel da Silva Neto, Airport, 59607-360 Mossoró-RN, Brazil; ^3^Department of Morphology, Bioscience Center, Federal University of Rio Grande do Norte, University Campus, Lagoa Nova, 59078-900 Natal, RN, Brazil; ^4^Laboratory of Neuroanatomy, Department of Morphology, Bioscience Center, Federal University of Rio Grande do Norte, University Campus, Lagoa Nova, 59078-900 Natal, RN, Brazil; ^5^Laboratory of Neurochemical Studies, Department of Physiology, Bioscience Center, Federal University of Rio Grande do Norte, University Campus, Lagoa Nova, 59078-900 Natal, RN, Brazil

## Abstract

A number of evidences show the influence of the growth of injured nerve fibers in peripheral nervous system as well as potential implant stem cells (SCs). The SCs implementation in the clinical field is promising and the understanding of proliferation and differentiation is essential. This study aimed to evaluate the plasticity of mesenchymal SCs from bone marrow of mice in the presence of culture medium conditioned with facial nerve explants and fibroblast growth factor-2 (FGF-2). The growth and morphology were assessed for over 72 hours. Quantitative phenotypic analysis was taken from the immunocytochemistry for glial fibrillary acidic protein (GFAP), protein OX-42 (OX-42), protein associated with microtubule MAP-2 (MAP-2), protein *β*-tubulin III (*β*-tubulin III), neuronal nuclear protein (NeuN), and neurofilament 200 (NF-200). Cells cultured with conditioned medium alone or combined with FGF-2 showed morphological features apparently similar at certain times to neurons and glia and a significant proliferative activity in groups 2 and 4. Cells cultivated only with conditioned medium acquired a glial phenotype. Cells cultured with FGF-2 and conditioned medium expressed GFAP, OX-42, MAP-2, *β*-tubulin III, NeuN, and NF-200. This study improves our understanding of the plasticity of mesenchymal cells and allows the search for better techniques with SCs.

## 1. Introduction

All the facial muscles are innervated by the motor division of the 7th cranial nerve, the facial nerve. Important physiological functions rely on the same integrity, such as tearing and eye protection, taste (the anterior two-thirds of the tongue), food intake (the orbicularis oris muscle, which takes part at the beginning of the process), and salivation[1]. There are many causes for their dysfunction, the most common of which are inflammatory processes [2]. Injury to peripheral nerves represents a challenge for the recovery of nerve function. Facial nerve functional recovery is dependent on new axon outgrowth, myelination, and correct reinnervation on the target organ [3].

Many models of peripheral nerve injuries have been treated with the combinations of cells and conduct. Furthermore the evaluation protocols are currently being researched [4]. The combination of transplanted cells and axon guide provides adequate support for neural regeneration and has been investigated as a strategy to overcome the limitations of surgical procedures [5]. In this universe, therapies with the use of stem cells (SCs) have been quite promising. These cells are capable of self-replication or manufacturing new cells of one or more lineages. Two specific types of SCs have been identified: embryonic stem cells and adult stem cells. The ideal cell should display a high level of proliferation, a good control of their proliferative activity, and phenotypic plasticity [6].

The control of plasticity is a complex event that requires the output of an undifferentiated cell to a given state stage of development. Cell determination is seen as a static event initiated and aided by intrinsic and extrinsic factors. The manipulation of environmental signals can induce cell differentiation in certain strains and may help in understanding the mechanisms of neural and glial development. It is still unclear what mechanisms govern the differentiation and migration of mesenchymal cells to injured areas; however, it is likely that the presence of neurotrophic factors, cytokines, and local stem cells has an influence. Understanding the environment in which these cells are cultured enables the understanding of the best methods of cell expansion and the creation of separate protocols of plasticity. Cells grown in a tissue are influenced by the organ that surrounds their environment. Once they are removed, they are free to take a different destination [7].

Mesenchymal cells are most commonly used in studies involving cell therapy. They are found in many tissues and represent an easily accessible source for autologous possible treatments. They are able to secrete neurotrophic factors and stimulate and support the growth, maturation, and differentiation of neural cells [8]. In this perspective, the mesenchymal cells could represent an alternative to the use of nervous stem cells. A culture of mesenchymal cells with a medium conditioned by facial nerves could recreate (at least partially) a microenvironment of peripheral nerves. Thus, the present study aimed to evaluate the morphological changes, growth, and plasticity of mesenchymal cells of a culture medium conditioned by explants of the facial nerve and the presence of FGF-2.

## 2. Materials and Methods

### 2.1. Animals

Male Wistar rats of approximately 250 g were used under the approval of the Ethics Committee on Animal Experimentation from the State University of Rio Grande do Norte (UERN), Protocol number 007/2012 in accordance with the ethical principles adopted by the Brazilian Society of Laboratory Animal Science and according to the law number 11,794, Arouca law, Ministry of Science, Technology and Innovation [9]. The animals were obtained from the vivarium of UERN. After the breastfeeding period, they were kept in a vivarium in separate cages with adequate housing conditions with free access to food and water until they were of appropriate weight and age.

### 2.2. Isolation and Culture of Bone Mesenchymal Stem Cells (BMSCs)

Mesenchymal SCs were collected from bone marrow of male three-month-old Wistar rats. The animals were euthanized with an overdose of anesthetic (Ketamine and Xylazine of Agener Union). The animals were dissected under aseptic conditions for removal of femurs and tibias. The freshly dissected bones were held in a conical tube with phosphate-buffered saline (PBS) until the removal of any portion of the muscle tissue attached to bone occurred. Under laminar flow, 60 mm culture plates were prepared with cell culture medium. The culture medium used was the low Knockout Dulbecco's modified Eagle's medium (DMEM) supplemented with 10% fetal bovine serum and 10 U/mL penicillin G, 10 *μ*g/mL streptomycin, and 25 *μ*g/mL amphotericin B (Gibco, USA).

Unattached cells and residual nonadherent red blood cells were removed after 24 hours of washing of PBS. The medium was changed at 72-hour intervals until the cells became confluent. After cells reached 90% confluence, they were trypsinized and subcultured at a density of 1 × 106 cells/plate. Cells were passaged repeatedly after achieving a density of 80–90% until reaching passage 1.

### 2.3. Conditioning DMEM for Facial Nerve Explants

Under laminar flow, 60 mm (P60) culture plates with lids were prepared with 5 mL of Leibovitz-15 medium (L15: Gibco, USA). In animals that underwent extraction of mesenchymal cells, the facial regions were shaved. Local asepsis was used with 2% chlorhexidine. Surgical approaches were made between the preauricular and perioral regions in both antimeres so that the buccal and mandibular branches were highlighted and isolated. Each segment was about 15 mm in length and 1 mm in diameter. The branches of the facial nerve were removed and placed in P60 with L15 medium under aseptic surgical technique with the aid of microdevices (scissors, forceps, and retractors). All of the excess tissues (muscle, fat, and blood vessels) attached to the nerves were removed under magnification by a SZ61 stereomicroscope (Olympus, Japan).

Next, the perineurium of the nerves was removed under magnification and microsurgical technique. The dissected nerves were segmented into explants of 1 mm length each. Under laminar flow, nerve fragments were placed in 60 mm plates with 1.5 mL of DMEM plus 10% fetal bovine serum and 0.1% gentamicin, with this medium called D-10. Excess medium was removed from the explants so that they would not be floating nor submerged in the medium. The D-10 medium from these cultures was changed two times per week, and the explants were transferred to a new plate with fresh medium 1 time per week. The medium was changed once and then disposed. This procedure allowed for an adequate nutrient supply to the explants and their reactivity. At this stage, the explants were removed to a new culture plate after five days, which led to the phasing out of the tissue cells that migrated to the bottom of the plate. On average, 18 explants were plated in each of the facial nerve P60 boards.

### 2.4. Neurogenic End Glial Differentiation

Bone marrow stem cells (BMSCs) were placed in 48 P60 and were observed in three time periods: 24, 48, and 72 hours. With this procedure, it was possible to assess the adhesion and proliferation of mesenchymal SCs from bone marrow into the following groups: group 1: BMSCs + DMEM, group 2: BMSCs + medium D-10, group 3: BMSCs + FGF-2, and group 4: BMSCs + medium + 10-D + FGF-2. In groups 3 and 4, 1 *μ*L of FGF-2 (Sigma, USA) of concentration 1 : 10 was added. In groups 2 and 4, 1 mL of medium (D-10) conditioned by facial nerve explants was added that had been transferred at least once to the other plate. The cell count was done using phase microscopy in 9 nonoverlapping fields at 10x magnification by a CKX41 microscope (Olympus, Japan). Photomicrographs of the 4 groups were performed during the periods of 24 hours, 48 hours, and 72 hours. The cell morphology was observed using phase contrast microscopy at 10x magnification. After 72 hours of cell observation, we proceeded to immunocytochemistry.

### 2.5. Immunofluorescence Staining

On the fourth day (96 hours), the cells had adhered to the plates, the medium was removed, and the cells were washed in two steps of five minutes in PBS 0.1 M, pH 7.4, fixed in paraformaldehyde (PFA) 4% for thirty minutes and washed again in three baths of PBS (five minutes each). Then, the cells were treated with 0.5% Triton (Sigma) for 10 minutes and washed in PBS. Subsequently, blocking of nonspecific sites was done for 30 minutes in a PBS 0.1 M solution containing 0.2% Triton and 1% cattle serum albumin (CSA). Plates were incubated for 2 hours at room temperature with anti-mouse GFAP (Sigma, 1 : 400), anti-mouse OX-42 (Millipore, 1 : 500), anti-mouse MAP-2 (Abcam, 1 : 2000), anti-rabbit *β*-tubulin III (Millipore, 1 : 500), anti-mouse NeuN (Millipore, 1 : 500), and anti-mouse NF-200 (Abcam, 1 : 1200).

Upon completion of this step, cells were washed in PBS (0.1 M, pH 7.4) for five minutes and incubated for 1 hour with anti-mouse and anti-rabbit secondary antibody produced in donkeys (Jackson, USA), conjugated to fluorophore AlexaFluor 488, AlexaFluor 594, FITC, and TRITC, and kept under refrigeration with absence of light. After secondary incubation, cells were washed with PBS for five minutes and immediately examined in a fluorescence microscope (Eclipse E200, Nikon) and later in another fluorescence microscope (Eclipse Ni, Nikon). Photomicrographs were made with Moticam 3.0 and 5.0 (Motic) digital cameras increased by 4x, 10x, and 20x in 9 fields in a predetermined sequence on each plate. The presence of fluorescent staining was recorded in mesenchymal cells, taking care to examine the subcellular, cytoplasmic, or nuclear compartment.

### 2.6. Statistical Analyses

Two independent investigators calibrated (kappa = 0.94) counted cells per field in absolute numbers, using cell cultures of at least 3 different experiments with 10x magnification. The Motic Images Plus 2.0 (Motic) software for morphological observation, the ImageJ software for cell count, and Adobe Photoshop CS6.0 (Adobe) software to fix minimum brightness and contrast of the photomicrographs were used. The database search was built on the SPSS platform software (Statistical Package for Social Sciences) version 21.0, with subsequent consistency check of typing. After the final structure of the database, a descriptive analysis of all data was initially performed. The data of cell expansion (number of cells) were statistically compared by analysis of variance (ANOVA) with the Bonferroni test and considered significant when *P* < 0.05.

## 3. Results

**Figure 1:**
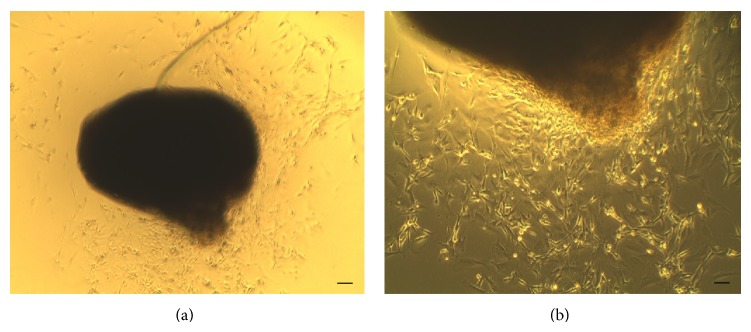
Facial nerve reactive explants. (a): 4x; (b): 10x. Scale: 100 *μ*m.

Through the methods employed, mesenchymal cells cultures were obtained essentially free of other cell types. In turn, the cells were well adhered to the bottom of the plate and exhibited polygonal morphology characteristics. To proliferate and reach confluence, these cultures were extremely juxtaposed with their individual definition, managing to form a monolayer. In the plates with facial nerve explants, there was a slight migration of fibroblastoid morphology of cells around the explants after 2 days of observation. The nerve segments remained reactive until the tenth day after cultivation (Figure 1). The following data refer to growth potential, morphological, and phenotypic changes and the results of three experiments on 48-culture P60 plates divided in four groups: group 1 or control group: BMSCs + DMEM, group 2: BMSCs + medium D-10, group 3: BMSCs + FGF-2, and group 4: BMSCs + medium + 10-D + FGF-2.

### 3.1. Morphology of BMSCs

**Figure 2:**
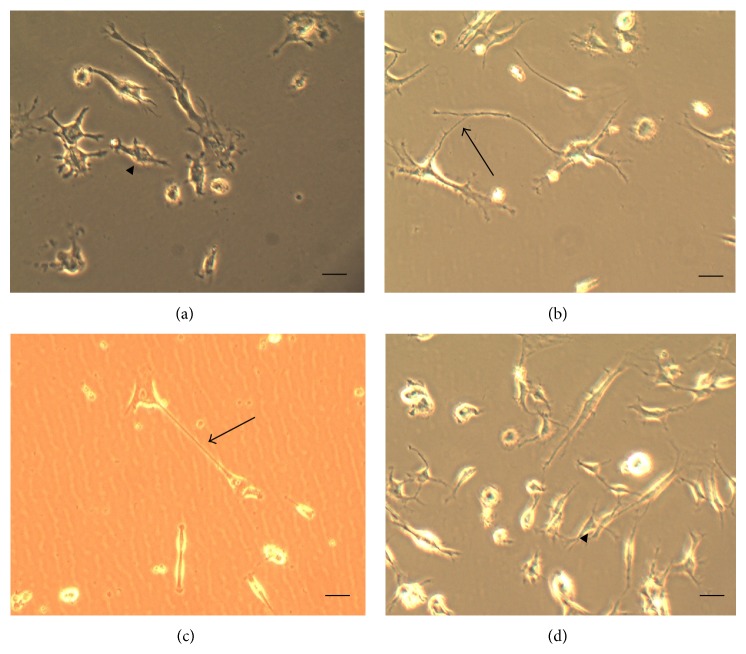
Morphological appearance of mesenchymal cells. (a) Bipolar cell and (b) cell with secondary prolongment, Group 2; (c) cell with primary prolongment and (d) tripolar cell, Group 4 (10x). Scale: 100 *μ*m.

**Figure 3:**
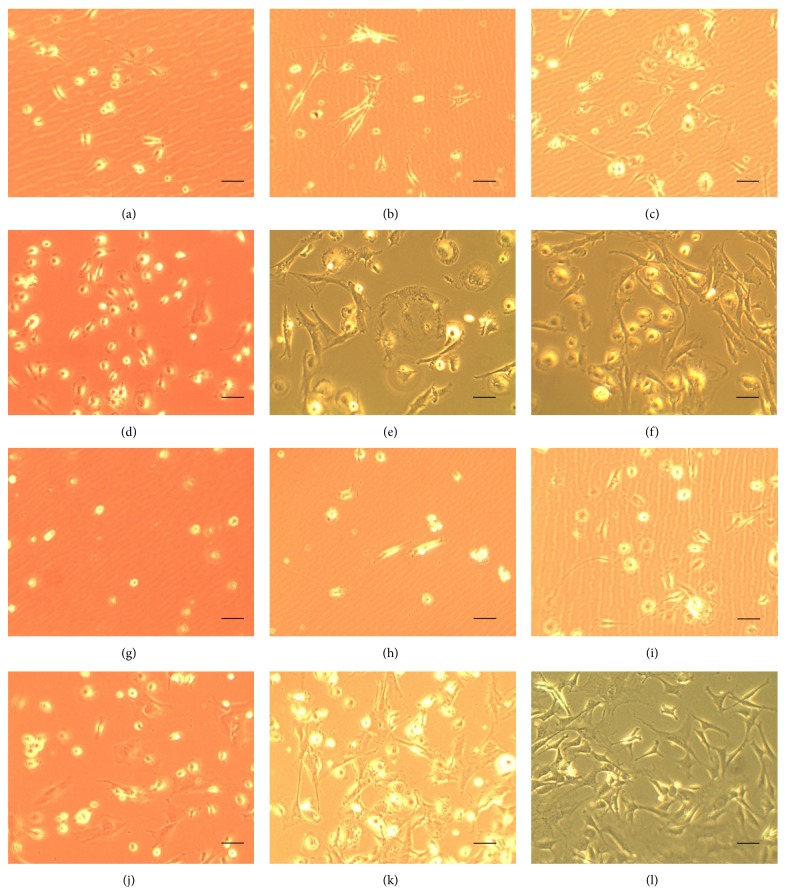
Morphology of mesenchymal cells in the experimental groups after observation for 72 hours. group 1: (a) 24 hours, (b) 48 hours, and (c) 72 hours; group 2: (d) 24 hours, (e) 48 hours, (f) and 72 hours; group 3: (g) 24 hours, (h) 48 hours, and (i) 72 hours; group 4: (j) 24 hours, (k) 48 hours, and (l) 72 hours. Scale: 100 *μ*m.

The populations of mesenchymal cells used were morphologically homogeneous. After exposure to the induction medium, conditioned mesenchymal cells of groups 2 and 4 showed very rapid morphological changes (after 6 hours). Most of the cells retracted their cytoplasm to form a spherical body of the cell and cellular projections issued. At the end of this process, it was possible to morphologically identify distinct subsets of mesenchymal cells, irrespective of their origin (groups 2 and 4) (Figure 2). Cultures of mesenchymal stem cells derived from bone marrow when cultured in a medium conditioned by the facial nerve and FGF-2 showed more noticeable changes when compared to the cultures grown only in D-10 medium and DMEM medium with or without FGF-2, especially on the third day of microscopic observation (Figure 3).

At 72 hours, it was found that the populations gradually multiplied from the first day until the last day of observation in all experimental groups, except in group 1 specifically, on the second day. In contrast, on day 2, 501 cells were counted in a single field of group 4. Accounting for all cells in absolute numbers over the three days, group 4 (7583) and group 2 (6422) stood out with the highest number of cells.

**Figure 4:**
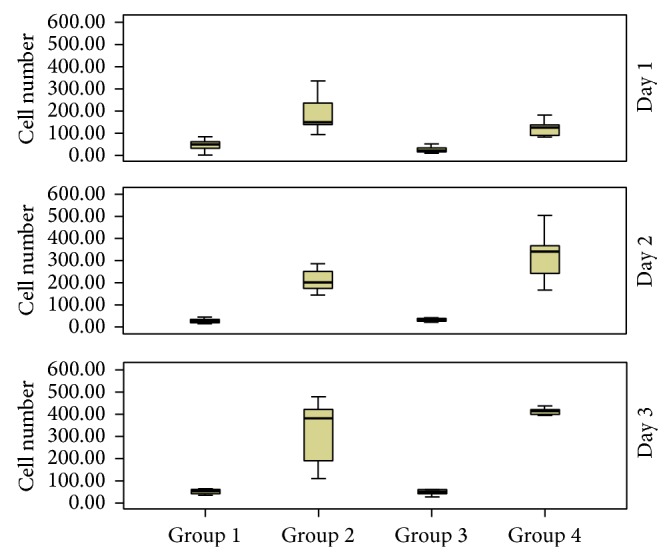
Number of mesenchymal cells observed on days 1, 2, and 3 in accordance with the experimental group. *P* values: a = 0.006, b = 0.0027, c = 0.001, d = 0.001 and = 0.001, f = 0.001, g = 0.002, h = 0.001, i = 0.001, j = 0.001, k = 0.001, l = 0.001, m = 0.001, and n = 0.001.

Comparing the mean number of cells observed in all groups by day log (days 1, 2, and 3), it was found that within 24 hours the average of counted cells was higher in group 2 than the number of cells in group 1 (Figure 4). The mean number of cells in group 4 was also higher than in group 1 (*P* = 0.001) or group 3 (*P* = 0.001) on day one. After 48 hours of induction, the average of mesenchymal cells observed in group 4 was higher than that of the other groups and the average of group 2 was also higher than that of group 1 (*P* = 0.001) or group 3 (*P* = 0.001) (Figure 4). On the last day of counting, the groups 2 and 4 did not differ in the number of cells (Figure 4).

### 3.2. Immunofluorescence Analysis

**Figure 5:**
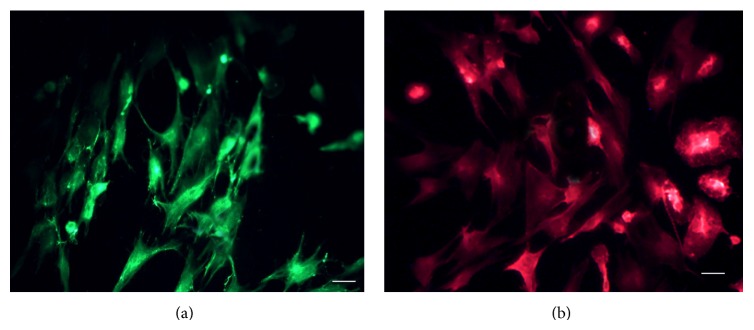
Mesenchymal cells of group 2 underwent immunoflorescence GFAP/Alexa 488 (a) and OX-42/TRITC (b) antibody (10x). Scale: 100 *μ*m.

After 72 hours of observation, we proceeded to the immunocytochemistry with the fluorescence microscope filters closed to validate the markup. Cells in groups 1 and 3 did not express any glial or neuronal protein, but in contrast the populations of group 2 expressed GFAP and OX-42 (Figure 5). The populations of cells in group 4 expressed GFAP, OX-42, MAP-2, *β*-III tubulin, NeuN, and NF-200 (Figure 5). Furthermore, the morphological changes of cells were present again and more frequent in group 4.

**Figure 6:**
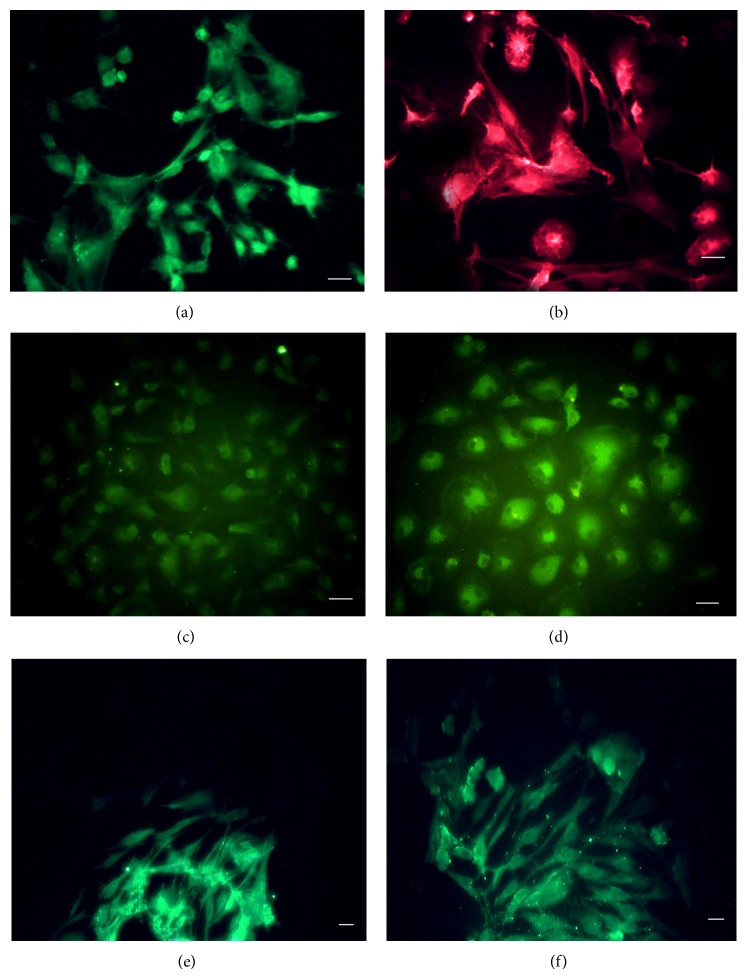
Mesenchymal cells of group 4 underwent immunofluorescent GFAP/Alexa 488 (a), OX-42/TRITC (b), MAP-2/FITC (c), *β*-tubulin III/FITC (d), NeuN/Alexa 488 (e), and NF-200/Alexa 488 (f) antibodies (10x). Scale: 100 *μ*m.

In group 2, there was a migration of GFAP and OX-42 immunoreactivity from the cytoplasm to the nuclear compartment of the cells and a higher intensity of the marking. It is still possible to observe the marking in the cytoplasmic compartment with a smaller and less wide (Figure 6(a)) intensity. In group 4, it was possible to locate several cells with a characteristic bipolar shape (Figures 6(e) and 6(f)), alongside cells with typical mesenchymal morphology. There was a larger and uniform marking of intensity throughout the cytoplasm of *β*-tubulin III (Figure 6(d)) and in nuclear compartments for OX-42 and MAP-2 (Figures 6(b) and 6(c)).

## 4. Discussion

The mesenchymal cells derived from bone marrow are seen as a promising source of stem cells for their accessibility, their proliferative potential, and their capacity of differentiation. These cells harvested from the marrow compartment of bone marrow were named bone marrow derived mesenchymal stem cells, as one of the earliest multipotent stem cells attracting researchers' attention [10]. Bone marrow contains two distinct populations of stem cells: hematopoietic stem cells and stromal progenitors. This is a primary site for MSCs from bone, cartilage, tendons, muscles, and adipose tissue. The potential of mesenchymal cells to form bone, cartilage, and adipose tissue in vivo and in vitro has been well documented. The techniques used for purification, expansion, and osteogenic, adipogenic, and chondrogenic differentiation of human mesenchymal cells can address the challenges of tissue engineering [11]. Mesenchymal cells, being multipotent, satisfy the requirement for transplanted cells ideally guarding quick, rapid expansion, and poor immunogenicity. Furthermore, they can differentiate into Schwann cells [12].

Studies have demonstrated that direct adhesion was the preferred method for isolating and purifying mesenchymal cells [13, 14]. The identification of mesenchymal cells was also primarily based on the morphological and functional characteristics. The images of cell cultures showed that the cells had a starry shape and were attached to the bottom of the plates. Similarly, cell adhesion and viability in DMEM and morphological appearance of the cells were used to characterize them as mesenchymal lineage in our study. In 2002, Kabos et al. [15] showed the first instance of a primary culture of mesenchymal cells differentiating in neurons and glia. These cells could be good candidates for the transplantation and repair in the nervous system because of their accessibility and their potential for expansion in vitro. Several studies have shown that the use of stem cells can replace the use of Schwann cells in an injured environment [16–19]. Their use in the surgical repair of peripheral nerves has improved axonal regeneration and functional recognition [20].

Recently, Salomone et al. [19] concluded that undifferentiated stem cells associated with nerve grafts or conduits of polyglycolic acid showed functional and morphological improvement in facial nerve trauma models. Similarly, Wang et al. [21] demonstrated that mesenchymal cells differentiated in rabbit Schwann cells were effective in regenerating facial nerve myelination and when combined with autologous venous conduits. The functional recovery of the facial nerve is dependent on the growth of new axons, myelination, and correct reinnervation of the target organ. In this study, the sectioned nerves treated with Schwann cells and veins were more effective in myelination of new regenerated axons.

In our study, 4 different culture media were used. Mesenchymal cells belonging to group 1 were cultured in DMEM alone. FGF-2 was added to DMEM in group 3 culture. In group 2, the DMEM was conditioned from explants of the facial nerve. Lastly, in group 4, a conditioned medium supplemented with FGF-2 was used. Our results showed that the mean number of cells counted after 24 hours in group 2 was higher than the number of cells from the other groups. On the second day after induction, the average of mesenchymal cells observed in group 4 was higher than the other groups, and, on the last day of counting, the groups 2 and 4 did not differ in the number of cells. In general, the proliferative activity found mainly in groups 2 and 4 shows an important characteristic of mesenchymal cells: rapid expansion in a short period of time.

Another important aspect found in our study was the morphological changes acquired by cells throughout the days. Cells grew at least in the conditioned medium (groups 2 and 4) showed similar morphological and faster changes at certain times with neurons and glial cells. The fibroblastoid and spindle factors were also identified. These changes were found in most cells of group 4. Kang et al. [22] investigated the neural differentiation of stem cells derived from muscle followed by treatment with an anticonvulsant and FGF. The morphological changes observed in cultures stimulated with FGF for 24 hours were consistent with GABA neurons. Moreover, the anticonvulsant and FGF treated cells were more similar to oligodendrocytes. Ying et al. [23] used both the epidermal growth factor combined and the epidermal growth factor not combined with the basic fibroblast growth factor BDNF to induce neuronal differentiation of adipocytes. After differentiation, the exhibited morphological changes were consistent with the formation of axonal and dendritic projections.

The most important factor for cell survival in a tissue receptor aspect is the microenvironment. Initially, this aspect is related to the expression of cell surface adhesion markers that interact with components of the extracellular matrix. Added to paracrine growth factors secreted by adjacent cells, the microenvironment effects provide conditions for their survival, migration, and tissue invasion and differentiation [24]. Currently, neuronal differentiation can be achieved in four ways: use of neurotrophic factors or cytokines, exposure to chemical inducers, or a combination of these cocultures of nerve cells [25].

In our research, the medium created from nerve fragments could simulate a situation of in vivo nerve injury. Thus, it was expected that the explants became reactive and naturally secrete factors that possibly try to regenerate nerves after trauma. The higher number of cells counted in groups 2 and 4 and the morphological changes are evident. The DMEM used as a control was very important to investigate whether the mesenchymal cells acquired distinct phenotypes not induced during the days. Because the properties of neurotrophic factors are quite discussed, the use of FGF-2 could enhance the phenotypic effects of cells and induce a higher proliferative activity. FGF-2 promotes neuronal survival and acts on the proliferation of SCs and interaction between glial cells and neurons.

Our experiments have demonstrated that cells from the groups 2 and 4 expressed GFAP and OX-42. GFAP is used as an astrocytic marker and can also be expressed in immature neural progenitor cells. OX-42 is used in dendritic cells, granulocytes, and microglia. In addition to these findings, cultivating the cells conditioned with a medium supplemented with FGF-2 also expressed MAP-2, *β*-tubulin III, NeuN, and NF-200. They are used to identify progenitor or mature neuronal cells. We hypothesize that the fibroblast growth factor enhanced the plasticity of cells in group 4. The expression of these antibodies in a short period of time after induction (4 days) can be justified by the apparent reactivity of nerve explants. The histological way the facial nerve is organized possibly explains the high reactivity as well the fast deposition of neurotrophic factors in the conditioned medium. The microenvironment of peripheral lesion simulated by a conditioned medium from explants of the facial nerve may explain the rapidity of neuronal and glial plasticity found.

Recently, Liu et al. [11] did neuronal induction in three cultures of mesenchymal cells and evaluated their findings among subcultures 3–5. MSCs were induced with DMEM/F12, FCS, and B27 supplement, 20 ng/mL of basic fibroblast growth factor, and epidermal growth of 20 g/mL factor. After 7 days, the medium was supplemented with retinoic acid and the cells were cultured for 7 days. The mesenchymal cells became thinner and had longer protrusions. Normal mesenchymal cells also expressed *β*-tubulin III, suggesting that these cells could retain the ability of neuronal differentiation. Similarly, Taha et al. [26] evaluated the effectiveness of a synthetic medium containing serum (KoSR) to differentiate into neuronal stem cells and embryonic stem cells derived from rat. Furthermore, it (KoSR) was also compared to the potential neuronal differentiation of stem cells derived from cultured adipocytes in a medium with low-serum condition (4% fetal bovine serum). A serum with synthetic effects of neurogenic factors (*β*-ME) was also examined in these two conditions. Their results demonstrated that neuronal differentiation of adipocytes stem cells can result in heterogeneous populations (dopaminergic neurons, GABA and cholinergic neurons). The induction medium supplementation with certain growth factors and differentiation appear to be necessary for the production of a specific neuronal population.

In summary, the isolated mesenchymal cells showed typical morphology and rapid expansion, especially the cells that were exposed to a medium conditioned by the facial nerve. Furthermore, some significant morphological characteristics similar to glial cells and neurons were present and the proliferative activity in groups 2 and 4 in a short period ensures their differentiation potential under in vitro exposure to appropriate stimuli. Additionally, BMSCs cultured with a conditioned medium acquired a phenotype consistent with a glial lineage (GFAP and OX-42). FGF-2 added to the conditioned medium potentiated this effect, so that the mesenchymal cells express neuronal proteins (MAP-2, *β*-III tubulin, NeuN, and NF-200) and glial protein (GFAP and OX-42). Although the phenotypic changes are consistent with neuronal and glial differentiation in mesenchymal cells from bone marrow, it cannot be said that these cells are indeed functional neurons or glial cells. Most neurophysiological studies are needed to confirm whether these differentiated cells function as neurons with respect to the development and maintenance of membrane potentials and action potentials, or as a useful framework in glial cells and regeneration of the nervous system.
